# Evaluation of GeneXpert MTB/RIF for Diagnosis of Tuberculous Meningitis

**DOI:** 10.1128/JCM.01834-13

**Published:** 2014-01

**Authors:** Nguyen Thi Quynh Nhu, Dorothee Heemskerk, Do Dang Anh Thu, Tran Thi Hong Chau, Nguyen Thi Hoang Mai, Ho Dang Trung Nghia, Pham Phu Loc, Dang Thi Minh Ha, Laura Merson, Tran Thi Van Thinh, Jeremy Day, Nguyen van Vinh Chau, Marcel Wolbers, Jeremy Farrar, Maxine Caws

**Affiliations:** a Oxford University Clinical Research Unit, Hospital for Tropical Diseases, Wellcome Trust Major Overseas Programme, Ho Chi Minh City, Vietnam; b Hospital for Tropical Diseases, Ho Chi Minh City, Vietnam; c Pham Ngoc Thach Hospital for Tuberculosis and Lung Diseases, Ho Chi Minh City, Vietnam

## Abstract

Tuberculous meningitis (TBM) is the most severe form of tuberculosis. Microbiological confirmation is rare, and treatment is often delayed, increasing mortality and morbidity. The GeneXpert MTB/RIF test was evaluated in a large cohort of patients with suspected tuberculous meningitis. Three hundred seventy-nine patients presenting with suspected tuberculous meningitis to the Hospital for Tropical Diseases, Ho Chi Minh City, Vietnam, between 17 April 2011 and 31 December 2012 were included in the study. Cerebrospinal fluid samples were tested by Ziehl-Neelsen smear, mycobacterial growth indicator tube (MGIT) culture, and Xpert MTB/RIF. Rifampin (RIF) resistance results by Xpert were confirmed by an MTBDR-Plus line probe assay and all positive cultures were tested by phenotypic MGIT drug susceptibility testing. Overall, 182/379 included patients (48.0%) were diagnosed with tuberculous meningitis. Sensitivities of Xpert, smear, and MGIT culture among patients diagnosed with TBM were 59.3% (108/182 [95% confidence interval {CI}, 51.8 to 66.5%]), 78.6% (143/182 [95% CI, 71.9 to 84.3%]) and 66.5% (121/182 [95% CI, 59.1 to 73.3%]), respectively. There was one false-positive Xpert MTB/RIF test (99.5% specificity). Four cases of RIF resistance (4/109; 3.7%) were identified by Xpert, of which 3 were confirmed to be multidrug-resistant (MDR) TBM and one was culture negative. Xpert MTB/RIF is a rapid and specific test for the diagnosis of tuberculous meningitis. The addition of a vortexing step to sample processing increased sensitivity for confirmed TBM by 20% (*P* = 0.04). Meticulous examination of a smear from a large volume of cerebrospinal fluid (CSF) remains the most sensitive technique but is not practical in most laboratories. The Xpert MTB/RIF represents a significant advance in the early diagnosis of this devastating condition.

## INTRODUCTION

Tuberculous meningitis (TBM) is the most devastating consequence of infection with Mycobacterium tuberculosis. Approximately a third of patients die soon after presenting to hospital, and many of those surviving are left with severe neurological sequelae (1, 2). In patients with HIV coinfection, mortality exceeds 60% (3). Early diagnosis and treatment for TBM have been shown in numerous studies to be the best predictor of survival (4–8). However, many patients are diagnosed late because initial signs are aspecific, and rapid and sensitive diagnostic tests are lacking. Many patients are initially treated empirically with broad-spectrum antibiotics until clinical deterioration warrants adjustment of the differential diagnosis (9). In low-resource settings, limited access to health care, limited diagnostic capacity, and economic constraints frustrate early treatment initiation. In high-resource settings, clinical suspicion is often low, and lack of recognition may lead to treatment delay. A microbiologically confirmed TBM diagnosis is rare in most laboratories. Ziehl-Neelsen (ZN) microscopy staining of cerebrospinal fluid (CSF) is the most widely applied rapid diagnostic technique; however, sensitivity for TBM rarely exceeds 20% (10). Previous work has shown that testing a large volume (>7 ml) of CSF and meticulously examining ZN slides for up to 30 min before recording a negative result improve smear and culture sensitivity significantly (11). However, in our experience, this time investment is not feasible in most busy routine diagnostic laboratories, where it has been shown increasing the examination time of sputum smears to just 10 min can increase case detection by up to 70% compared with examination times routinely applied (12–14). Liquid culture techniques, including the mycobacterial growth indicator tube (MGIT; Bactec) and the mycobacterial observation drug susceptibility assay (MODS) culture offer improved sensitivity over solid culture, to a sensitivity of almost 60% (15). The clinical value of culture techniques is limited to diagnostic confirmation and drug susceptibility testing, because they take 1 to 4 weeks to return a positive result, and negative results cannot be used to exclude a TBM diagnosis. In addition, molecular typing of M. tuberculosis isolates can provide insights into epidemiology and immunopathogenesis.

Studies to identify useful biomarkers for TBM in CSF and blood are ongoing. Tests such as the adenosine deaminase assay (ADA) have been evaluated and may be used as an aid in diagnosis; however, they are not specific enough to differentiate TB meningitis from other forms of bacterial meningitis (16, 17).

A meta-analysis of nucleic acid amplification techniques (NAAT) showed wide variability for performance of in-house tests and sensitivity for commercial tests of below 60% (18). Individual reports of the use of in-house PCR have reported higher sensitivities, particularly with multiplex PCR techniques; however, these tests can be difficult to implement with appropriately rigorous quality controls in resource-limited, high-burden health care centers, where the need is greatest (16).

The GeneXpert MTB/RIF test (Cepheid) is a closed-cartridge-based system that is easy to operate by minimally trained staff and gives results in approximately 2 h (19). The Xpert MTB/RIF test was approved by the WHO in 2010 for the diagnosis of pulmonary TB following extensive evaluation projects in six countries led by the Foundation for Innovative New Diagnostics (FIND) (20).

The test is based on a real-time heminested PCR test which detects the presence of M. tuberculosis complex bacilli (21). By using 5 molecular beacons which span the *rpoB* gene 81-bp rifampin resistance-determining region (RRDR), the test simultaneously determines susceptibility to rifampin, which can be used as a surrogate marker for multidrug resistance (MDR) (21). The closed-cartridge system makes it possible for the assay to be used outside the laboratory environment, and studies assessing biosafety have suggested that the use of Xpert MTB/RIF carries a smaller biohazard risk than smear microscopy (19). The risk of cross-contamination is also reduced with the closed cartridge system (19). The test has shown a sensitivity above 90% for culture-positive tuberculosis, with high specificity in sputum samples. Sensitivity in individuals with HIV coinfection is over 80% (22–24). A recent Cochrane review concluded that the Xpert MTB/RIF as an initial replacement for sputum smear showed a pooled sensitivity of 88% (95% credible interval [CrI], 83 to 92%) and a pooled specificity of 98% (95% CrI, 97 to 99%) (25).

Several studies have reported successful use of the Xpert MTB/RIF test on extrapulmonary samples, with overall sensitivities of over 80% and specificity reaching 100% (26–30). However, the number of CSF samples in these studies combined was low, including only a total of 62 specimens. Due to the urgency of diagnosis in suspected TBM cases because of a rapid decrease of survival chances with the increase of severity (mortality for grade 1 patients is approximately 20%; for grade 3 it reaches 55% [31]), a rapid, accurate diagnostic test which also is able to identify rifampin resistance could have a great impact on survival.

The aim of the present study was to prospectively determine the diagnostic accuracy of Xpert MTB/RIF in a large consecutive series of samples from patients presenting to the Hospital for Tropical Diseases, Ho Chi Minh City, Vietnam, with suspected TBM. A preliminary review of the data in September 2011 resulted in a minor modification of the sample processing, with the addition of a brief vortexing step after addition of sample reagent.

## MATERIALS AND METHODS

### Ethical approval.

Ethical approval for this study was obtained from the Ethical Review Board of the Hospital for Tropical Diseases, Ho Chi Minh City, Vietnam, and from the Oxford Tropical Research Ethics Committee (OxTREC).

All adult patients (>18 years) presenting to the Hospital for Tropical Diseases (HTD), Ho Chi Minh City, Vietnam between 17 April 2011 and 31 December 2012 with suspected TBM and who underwent lumbar puncture as part of screening for enrollment in a randomized controlled trial of intensified treatment for TBM were included in the study. The full protocol of that trial is reported elsewhere (International Standard Randomized Controlled Trial Number ISRCTN61649292) (32). At HTD, clinicians are encouraged to draw at least 8 ml of CSF when possible, in order to improve microbiological confirmation rates (11). Approximately 1 ml of sample is sent to microbiology and biochemistry laboratories, and the remainder is sent to the TB laboratory. Upon receipt in the TB laboratory, CSF samples were centrifuged at 4,000 × *g* for 15 min. Supernatant was removed to leave a 0.5-ml deposit, which was then used for Ziehl-Neelsen smear preparation (100 μl), inoculation of MGIT culture (100 μl), and Xpert testing (200 μl). The remaining deposit was stored at −20°C. All tests were performed by one of three technicians highly experienced in microbiological tests for TBM diagnosis. Clinical data and results of biochemical investigations were not available to the technicians at the time of the test; technicians were aware of smear results.

### Ziehl-Neelsen smear.

Ziehl-Neelsen smears were prepared using standard methods with two modifications. First, the smear was layered, with two drops of CSF deposit applied. The layered smear was then stained according to standard procedures. Second, the ZN smear was meticulously examined for up to 30 min under a ×1,000 magnification before being recorded as negative. Observation of a single acid-fast bacillus was considered a positive result (11).

### Xpert MTB/RIF.

A 200-μl portion of the deposit was resuspended in phosphate-buffered saline to a 500-μl volume. The sample reagent supplied with the test (1.5 ml) was then added. Prior to August 2011, the mixture was then shaken by hand according to test instructions. Following a preliminary review of the Xpert data from 1 August, the mixture was vortexed for 30 s to ensure all bacteria were resuspended. The sample was left to stand for 15 min, as per the manufacturer's instructions, with intermittent manual shaking. The solution was then transferred to the Xpert cartridge using a Pasteur pipette, and the cartridge was loaded onto the Xpert machine for analysis. Results are reported as positive or negative for M. tuberculosis. Positive results were placed in one of four categories; very low, low, medium, or high. Rifampin resistance results were reported as susceptible or resistant.

### MGIT culture.

A 100-μl portion of the deposit was used to inoculate a MGIT tube containing 0.8 ml MGIT supplement (PANTA antibiotics [polymyxin B, amphotericin B, nalidixic acid, trimethoprim, and azlocillin] and growth supplements). MGIT tubes were incubated in a MGIT 960 machine until they were automatically identified as positive or for 56 days. All positive cultures were tested for susceptibility to rifampin, isoniazid, streptomycin, and ethambutol using a Bactec MGIT SIRE kit (Becton, Dickinson) according to the manufacturer's instructions (33).

### Line probe assay.

Cases of rifampin resistance detected by Xpert were confirmed using the MTBDRplus line probe assay (Hain Lifesciences, Germany) (34) on DNA extracted from a positive MGIT culture isolated from the same CSF sample. DNA was extracted from positive MGIT cultures using the cetyltrimethylammonium bromide (CTAB) method (35), and the purified DNA was then used for the MTBDRplus test using the manufacturer's instructions (36).

### Other investigations.

All patients underwent routine investigations for diagnosis of meningitis, including CSF biochemistry, cell counts, India ink stain for fungi, Gram stain, culture (on blood, chocolate, MacConkey, and Sabouraud dextrose agar), viral PCR (for herpes simplex virus [HSV] and varicella zoster virus [VZV]), and IgM and IgG serology for Japanese encephalitis (JE).

### Diagnostic classification.

For this study, patients were classified as having TBM if no other diagnosis was made and the attending physician made the decision to treat for TBM based on the clinical algorithm in Table 1. In addition, patients diagnosed with TBM were classified as having definite, probable, or possible TBM using this standardized case definition (37). Xpert MTB/RIF results were not included in the case definition, because it was the test under evaluation. Definite TBM was defined as a clinical syndrome consistent with TBM, with acid-fast bacilli seen on CSF smear or M. tuberculosis isolated in CSF MGIT culture. Patients in the “probable TBM” group had a diagnostic score of 10 or more without cerebral imaging (MRI or CT scan) or 12 or more with cerebral imaging, with at least 2 points from CSF or cerebral imaging criteria. Patients in the “possible TBM” group had a diagnostic score of between 6 and 9 if cerebral imaging was not performed or between 6 and 11 if cerebral imaging was performed (37). All patients who did not meet the criteria or did not receive treatment for TBM and received an alternative discharge diagnosis were classified as not having TBM.

**TABLE 1:** Clinical case definition^*a*^

Category (maximum category score)	Criterion	Diagnostic score
Clinical criteria (6)	Symptom duration of more than 5 days	4
	Systemic symptoms suggestive of tuberculosis (one or more of the following): wt loss (or poor wt gain in children), night sweats, or persistent cough for more than 2 weeks	2
	History of recent (within the past year) close contact with an individual with pulmonary tuberculosis or a positive TST or IGRA (only in children <10 years of age)	2
	Focal neurological deficit (excluding cranial nerve palsies)	1
	Cranial nerve palsy	1
	Altered consciousness	1
CSF criteria (4)	Clear appearance	1
	Presence of 10–500 cells per μl	1
	Lymphocytic predominance (>50%)	1
	Protein concn greater than 1 g/liter	1
	CSF to plasma glucose ratio of less than 50% or an absolute CSF glucose concn less than 2.2 mmol/liter	1
Cerebral imaging criteria (6)	Hydrocephalus	1
	Basal meningeal enhancement	2
	Tuberculoma	2
	Infarct	1
	Precontrast basal hyperdensity	2
Evidence of tuberculosis elsewhere (4)	Chest radiograph suggestive of active tuberculosis: signs of tuberculosis = 2; miliary tuberculosis = 4	2/4
	CT/MRI/ultrasound evidence for tuberculosis outside the CNS	2
	AFB identified or Mycobacterium tuberculosis cultured from another source—i.e., sputum, lymph node, gastric washing, urine, blood culture	4
	Positive commercial M. tuberculosis NAAT from extraneural specimen	4
Exclusion of alternative diagnoses	An alternative diagnosis must be confirmed microbiologically (by stain, culture, or NAAT when appropriate), serologically (e.g., syphilis), or histopathologically (e.g., lymphoma); the list of alternative diagnoses that should be considered, dependent upon age, immune status, and geographical region, include pyogenic bacterial meningitis, cryptococcal meningitis, syphilitic meningitis, viral meningoencephalitis, cerebral malaria, parasitic or eosinophilic meningitis (Angiostrongylus cantonensis, Gnathostoma spinigerum, toxocariasis, cysticercosis), cerebral toxoplasmosis and bacterial brain abscess (space-occupying lesion on cerebral imaging), and malignancy (e.g., lymphoma)	The individual points for each criterion (one, two, or four points) were determined by consensus and by considering their quantified diagnostic value as defined in studies.
Clinical entry criteria	Symptoms and signs of meningitis including one or more of the following: headache, irritability, vomiting, fever, neck stiffness, convulsions, focal neurological deficits, altered consciousness, or lethargy	
Tuberculous meningitis classification		
Definite tuberculous meningitis (patients should meet one set of criteria)	Clinical entry criteria plus one or more of the following: acid-fast bacilli seen in the CSF, M. tuberculosis cultured from the CSF, or a CSF positive commercial nucleic acid amplification test	
	Acid-fast bacilli seen in the context of histological changes consistent with tuberculosis in the brain or spinal cord with suggestive symptoms or signs and CSF changes, or visible meningitis (on autopsy)	
Probable tuberculous meningitis	Clinical entry criteria plus a total diagnostic score of 10 or more points (when cerebral imaging is not available) or 12 or more points (when cerebral imaging is available) plus exclusion of alternative diagnoses; at least 2 points should come from either CSF or cerebral imaging criteria	
Possible tuberculous meningitis	Clinical entry criteria plus a total diagnostic score of 6–9 points (when cerebral imaging is not available) or 6–11 points (when cerebral imaging is available) plus exclusion of alternative diagnoses; possible tuberculosis cannot be diagnosed or excluded without doing a lumbar puncture or cerebral imaging	
Not tuberculous meningitis	Alternative diagnosis established, without a definitive diagnosis of tuberculous meningitis or other convincing signs of dual disease	

a Modified with permission from reference 3. CNS, central nervous system; TST, tuberculin skin test; IGRA, interferon-gamma release assay; NAAT, nucleic acid amplification test; AFB, acid-fast bacilli; CT, computed tomography; MRI, magnetic resonance imaging.

### Statistical analysis.

Sensitivity, specificity, positive predictive value, and negative predictive value with 95% confidence intervals were calculated. The proportion of positive results for each test (smear, MGIT culture, and Xpert MTB/RIF) was compared using McNemar's test for paired samples. To determine if the introduction of a vortexing step after addition of the sample reagent altered sensitivity of the Xpert test, we also analyzed sensitivity and specificity in samples processed before and after 1 August 2011. The sensitivity of Xpert MTB/RIF stratified by CSF volume was also analyzed.

All statistical analyses were done using R version 2.15.1 (The R foundation for Statistical Computing) with the package epiR.

## RESULTS

**FIG 1:**
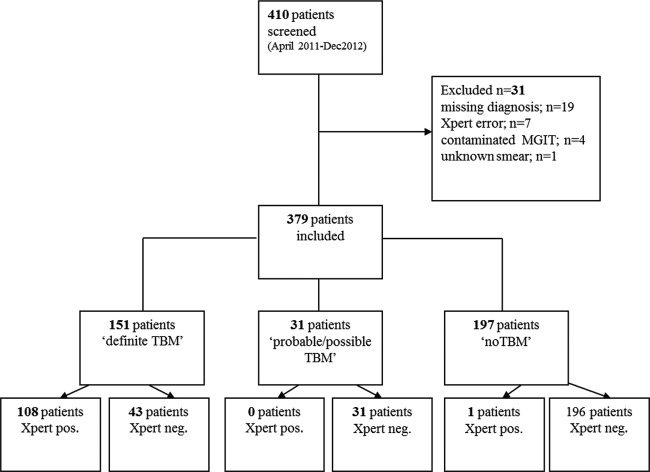
Flow chart of diagnosis for patients included in the study, showing final TBM diagnosis and Xpert MTB/RIF results. Neg., negative; pos., positive.

A total of 410 patients presented to the Hospital for Tropical Diseases with suspected TBM during the study period. A total of 31 patients were excluded: 19 for whom no final diagnosis could be made or clinical information was missing, 7 with an Xpert “error” result, 4 with contaminated cultures, and 1 with an unknown smear result. Thus, 379 eligible patients were included in the analysis. Of these, 151 were finally classified as having definite TBM, 18 as having probable TBM, 13 as having possible TBM, and 197 as not having TBM (Fig. 1).

Patients in the “not TBM” group were diagnosed with viral meningoencephalitis (*n* = 95), bacterial meningitis (*n* = 41), eosinophilic meningitis (*n* = 15), cerebral vascular event (*n* = 12), cryptococcal meningitis (*n* = 10), sepsis (*n* = 9), pneumonia (*n* = 7), cerebral toxoplasmosis (*n* = 2), psychiatric disorder (*n* = 1), cerebral tumor (*n* = 1), prolonged fever of unknown origin (*n* = 1), progressive multifocal leukoencephalopathy (*n* = 1), dengue (*n* = 1), and cerebral abscess (*n* = 1).

Overall, 79/379 (20.8%) patients were HIV infected, 108 (28.5%) were not HIV infected, and 192 (50.7%) had an unknown HIV status (i.e., they declined consent to an HIV test or were discharged before testing).

Of those classified as having definite, probable, or possible TBM (*n* = 182), 66 (36.3%) were HIV infected, 94 (52.6%) were not HIV infected, and 22 (12.1%) had an unknown HIV status.

### Diagnostic accuracy for TBM.

Overall, the sensitivity of Xpert was 59.3% (108/182; 95% confidence interval [CI], 51.8 to 66.5) compared to clinical diagnosis of TBM (definite, probable, and possible TBM). Specificity was 99.5% (95% CI, 97.2 to 100).

The sensitivity of smear relative to final clinical diagnosis was 78.6% (143/182 [95% CI, 71.9 to 84.3]), and that of MGIT culture was 66.5% (121/182 [95% CI, 59.1 to 73.3]) (Table 2). Since smear and MGIT culture were the reference microbiological tests for diagnosis of TBM, specificity of these tests could not be determined; however, all patients positive by smear or MGIT had a clinical picture consistent with TBM and had no other organisms isolated from the CSF.

**TABLE 2:** Results of smear, MGIT culture, and Xpert MTB/RIF testing by final diagnosis

Test	Result	No. (%)		
		TBM	Not TBM	Total
Xpert MTB/RIF	Positive	108 (59.3)	1 (0.5)	109
	Negative	74 (40.6)	196 (99.5)	270
	Total	182 (100)	197 (100)	379
Ziehl-Neelsen smear	Positive	143 (78.6)	0	143
	Negative	39 (21.4)	197 (100)	236
	Total	182 (100)	197 (100)	379
MGIT culture	Positive	121 (66.5)	0	121
	Negative	61 (33.5)	197 (100)	258
	Total	182 (100)	197 (100)	379

The sensitivity of Xpert MTB/RIF relative to smear was 73.4% (105/143 [95% CI, 65.4 to 80.5]), and that relative to MGIT sensitivity was 85.1% (103/121 [95% CI, 77.5 to 90.9]).

The sensitivity of Xpert MTB/RIF relative to clinical diagnosis was significantly lower than the sensitivity of smear relative to clinical diagnosis (−19.3%; *P* < 0.001) and slightly lower than that of MGIT culture relative to clinical diagnosis (−7.2%; *P* = 0.024). The sensitivities of smear and MGIT culture were also significantly different (−12.1%; *P* < 0.001).

The positive and negative predictive value of Xpert against final clinical diagnosis of TBM were 99.1% (108/109 [95% CI, 95.0 to 100]) and 72.5% (196/270 [95% CI, 66.9 to 77.8]), respectively.

### Diagnostic accuracy with addition of a vortexing step.

Prior to 1 August 2011, there were 48 patients included in the study, 26 of whom were finally diagnosed with TBM and 22 diagnosed as not having TBM. The sensitivity of Xpert for these samples was 50.0% (13/26 [95% CI, 29.9 to 70.1]), and that of smear was 88.5% (23/26 [95% CI, 69.8 to 97.6]). MGIT culture had a sensitivity of 57.7% (15/26 [95% CI, 36.9 to 76.6]). The sensitivity of Xpert for the “definite TBM” result was 54.2% (13/24 [95% CI, 32.8 to 74.4]).

After the introduction of the vortexing step on 1 August 2011, 331 patients were included in the study. Of these, 156 were finally classified as TBM cases and 175 as not having TBM. The sensitivity of Xpert for these samples was 60.9% (95/156 [95% CI, 52.8 to 68.6]), and the sensitivities of smear and MGIT, respectively, were 76.9% (120/157 [95% CI, 69.5 to 83.3]) and 67.9% (106/156 [95% CI, 60.0 to 75.2]). The sensitivity of Xpert for the “definite TBM” result was 74.8% (95/127 [95% CI, 66.3 to 82.1]).

The increase in sensitivity of Xpert for the diagnosis of definite TBM with the addition of the vortexing step to sample processing was 20.6% (*P* = 0.04).

### Diagnostic accuracy of Xpert MTB/RIF by CSF volume.

**FIG 2:**
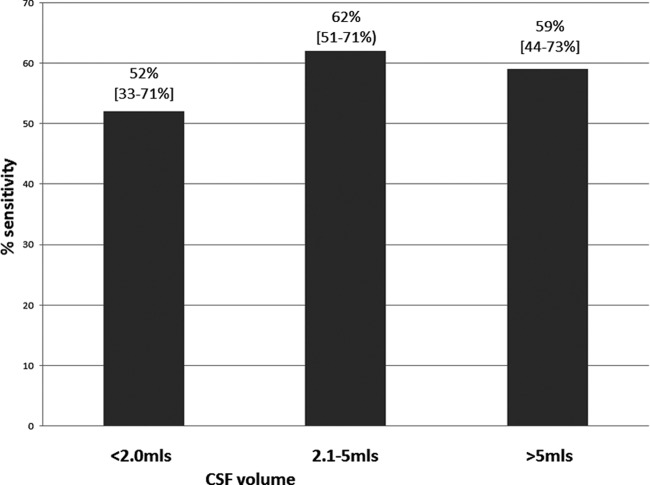
Sensitivity of Xpert MTB/RIF by volume of CSF processed for TB testing.

The volume of CSF received in the TB laboratory was recorded. Of all 379 CSF samples received, 65 (17.2%) were low volume (≤2.0 ml), 230 (60.6%) were medium volume (2.1 to 5.0 ml), and 84 (22.2%) samples were high volume (>5 ml). The sensitivities of Xpert MTB/RIF were 51.7% (15/29) (95% CI, 32.5 to 70.6) for low-volume samples, 61.5% (64/104) (95% CI, 44.2 to 73.0) for medium-volume samples, and 59.2% (29/49) (95% CI, 44.2 to 73.0) for high-volume samples (Fig. 2). Although the sensitivities for medium- and high-volume samples were greater than those for low-volume samples, this difference did not reach statistical significance (*P* = 0.341).

### Qualitative estimation of bacterial load.

The majority of Xpert results were categorized by Xpert as very low (54/109; 49.5%) or low (46/109; 42.2%), with 9 medium results (8.3%). Xpert did not report a high bacterial load for any CSF sample.

### Diagnostic accuracy by HIV status.

Sensitivity of Xpert for TBM against clinical diagnosis was significantly higher for HIV-infected patients (odds ratio = 4.01 [95% CI, 3.65 to 4.36; *P* < 0.001]) than for non-HIV-infected patients.

**FIG 3:**
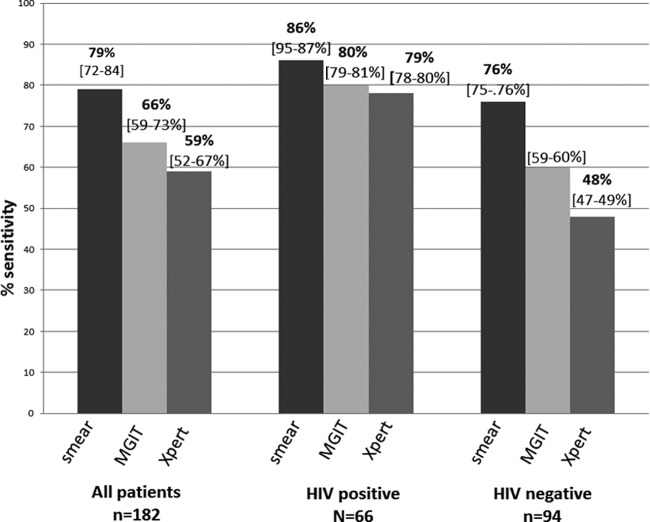
Sensitivities of ZN smear, MGIT culture, and Xpert MTB/RIF against the clinical gold standard for the diagnosis of TB meningitis in all patients and by HIV status. Values in brackets are 95% confidence intervals.

Among HIV patients, sensitivity was 78.8% (52/66 [95% CI, 77.6 to 79.7]), while it was 47.9% (45/94 [95% CI, 47.0 to 48.7]) in non-HIV-infected patients (Fig. 3).

### Detection of rifampin resistance.

Rifampin resistance was detected in four cases during the study by Xpert MTB/RIF. In three cases, the result was confirmed to be MDR TBM by an MTBDRplus line probe assay performed on DNA extracted from a positive MGIT culture. One case did not have a positive MGIT culture result. Xpert testing for rifampin resistance showed an “indeterminate” result in two cases. In one case, rifampin resistance was detected using the MGIT SIRE kit. Overall, phenotypic drug resistance testing of all MGIT-positive cultures using the MGIT SIRE kit showed 104 rifampin (RIF)-susceptible results and 5 RIF-resistant cases, 3 of which were detected by Xpert MTB/RIF.

However, it is not possible to draw robust conclusions about the sensitivity of Xpert for the diagnosis of MDR TBM given the low prevalence of MDR TBM in this study.

## DISCUSSION

We have shown that Xpert MTB/RIF is a rapid, specific test for the diagnosis of TBM. As with other tests for TBM, a negative result cannot exclude a diagnosis of TBM. While smear microscopy is a more sensitive test in our laboratory, this exceptional sensitivity compared to contemporary published reports from other laboratories is consistent with early reports of TBM diagnosis using smear (38) and previous publications from our laboratory. We believe that this exceptional sensitivity depends upon the meticulous examination of individual slides for 30 min by a highly skilled and experienced technician. This may be difficult to replicate outside a dedicated research setting due to the work burden in public health laboratories of resource-limited countries (11). The lower sensitivity of Xpert MTB/RIF compared to this meticulous smear process raises an important question regarding the inability of Xpert to detect bacilli visualized on the slide in some samples. Generally it is accepted that the lower limit of detection for standard sputum smear is around 10,000 CFU/ml, whereas that of Xpert MTB/RIF is reported to be 100 CFU/ml. PCR inhibitors in the CSF are unlikely to be the culprit; the Xpert MTB/RIF test contains an internal processing and amplification control (Bacillus atrophaeus subsp. globigii spores) which should lead to an error result if inhibitors are present in the sample. An alternative explanation may be the fact that unlike nonautomated PCR tests, the Xpert MTB/RIF depends upon capture of intact bacilli from the sample within the cartridge, and it is probable given the reported limits of detection that not all bacilli are captured and lysed during the process. Therefore, in high-volume laboratories with low sensitivity for CSF smear microscopy, Xpert MTB/RIF is likely to substantially improve the diagnostic confirmation of TBM, since it is less dependent on the skill and time of individual technicians.

A limitation of this study regarding the comparison of the sensitivities of Xpert MTB/RIF and MGIT culture is the difference in volumes of CSF deposit used for each test (200 μl for Xpert versus 100 μl for MGIT), which is likely to have decreased the sensitivity of MGIT culture in comparison with Xpert MTB/RIF. However, MGIT culture is not directly useful in making a decision to treat for TBM due to the time required for a positive result; TBM is a medical emergency, and delayed treatment is strongly associated with mortality in every case series. Further comparative study of the optimal sampling processing and inoculation volumes for each test to maximize early diagnosis while also obtaining M. tuberculosis isolates for drug susceptibility testing (DST) is required.

The sensitivity of Xpert reported here is similar to the sensitivity of other molecular techniques for TBM diagnosis. Xpert has two significant advantages: the closed-cartridge-based format and the ability to simultaneously detect M. tuberculosis and RIF resistance. The cartridge-based format removes the need for manual DNA extraction processing, and the closed system dramatically reduces any potential for cross-contamination of samples with PCR amplicons. The addition of a brief vortexing step after addition of the sample reagent improved sensitivity of Xpert in these paucibacillary samples, and further optimization of sample processing for extrapulmonary samples may be required to improve detection rates. The overall increase in sensitivity for TBM was 10%, with a 20% increase for definite TBM cases (*P* = 0.04).

The Xpert test system depends upon capture and lysis of whole bacilli (21), and therefore, as for other microbiological tests for TBM, high volumes (>7 ml) of CSF are crucial to achieving high sensitivity (11). Bacterial loads are higher in HIV-infected TBM patients, and this is reflected in the higher sensitivity for HIV-associated TBM of all the tests (Fig. 2); therefore, settings with a lower HIV prevalence among TBM patients will have correspondingly lower TBM confirmation rates. This is the inverse of the situation with pulmonary TB, where HIV-positive individuals with TB are less likely to be smear positive.

The costs of smear microscopy are substantially lower than the costs of an Xpert MTB/RIF test (consumable and reagent costs, approximately $2 [U.S. dollars] versus $15), but the hands-on time required to achieve high sensitivity in smear testing is greater (approximately 40 min for smear versus 20 min for Xpert). Additionally, in four cases, Xpert detected rifampin resistance within 2.5 h; we were unable to confirm rifampin resistance in one of these cases due to negative culture. Rapid detection of drug resistance in the paucibacillary CSF has been a major challenge to improving outcome for patients with MDR TBM. Without rapid diagnosis and administration of second-line regimens, mortality is 100% (39). However, rare false-positive results for rifampin resistance have been reported with Xpert (40), and the consequences of mistakenly treating a patient with rifampin-susceptible TBM with weak second-line regimens would be grave. It will be extremely difficult to accumulate sufficient data on MDR TBM diagnosis to demonstrate robustly the accuracy of the test for this condition due to its rarity, and accuracy must be inferred from other paucibacillary forms of TB. Therefore, a rifampin-resistant TBM diagnosis by Xpert should be evaluated in the context of the clinical information and response to treatment and, wherever possible, should be confirmed by a second rapid test, such as a line probe assay. An M. tuberculosis isolate remains necessary to confirm susceptibility patterns for all drugs, including rifampin, since Xpert detects *rpoB* mutations, which are present in only 95% of phenotypically rifampin-resistant M. tuberculosis isolates (41). Liquid culture methods, where available, have the highest sensitivity and speed for M. tuberculosis isolation (42). However, for patients with rifampin resistance detected by Xpert MTB/RIF and a clinical suspicion of MDR TBM, second-line drugs with appropriate CSF penetration should not be withheld until the results from conventional DST become available.

One patient in our cohort had a false-positive result for M. tuberculosis detection. This specificity is consistent with results reported for pulmonary TB. The patient was diagnosed with viral meningoencephalitis and did not meet the clinical criteria for TBM (scoring three points for the published case definition; with cranial imaging available, the minimum score required for a TBM diagnosis is six points). The patient was treated with antiviral drugs and antibiotics, but not with antimycobacterial drugs, and made a full recovery. The patient was still alive and well when contacted 10 months after presentation (9 July 2013). Without treatment, TBM is invariably fatal; therefore, the patient could not have had TBM.

In conclusion, the Xpert MTB/RIF test is able to rapidly confirm a diagnosis of TBM with 59% sensitivity and 99% specificity when large volumes of concentrated CSF and an additional vortexing step are used. This represents a significant advance in the early diagnosis of this devastating condition.
